# Therapy of IBS: Is a Low FODMAP Diet the Answer?

**DOI:** 10.3389/fpsyt.2020.00865

**Published:** 2020-08-31

**Authors:** Lauren P. Manning, C. K. Yao, Jessica R. Biesiekierski

**Affiliations:** ^1^Department of Rehabilitation, Nutrition and Sport, La Trobe University, Melbourne, VIC, Australia; ^2^Department of Gastroenterology, Central Clinical School, Monash University & Alfred Health, Melbourne, VIC, Australia

**Keywords:** FODMAP, dietary therapy, symptom management, IBS (irritable bowel syndrome), gut-brain axis

## Abstract

Irritable bowel syndrome (IBS) is the most prevalent functional gastrointestinal disorder with a worldwide prevalence of 11%. It is characterized by abdominal pain and altered bowel habits in the absence of underlying unique pathology. The condition is associated with poor quality of life and high use of healthcare resources required for management. The low FODMAP diet (LFD) is a recognized treatment for symptom management of IBS; however, approximately 30% of patients do not respond. The aim of this review was to understand the effectiveness and application of the LFD compared with other dietary and non-dietary interventions. Ten studies were included, eight of which assessed the LFD against other dietary interventions including traditional dietary advice, modified National Institute for Health and Care Excellence guidelines, a high FODMAP diet, gluten-free diet and Mediterranean diet, generalized dietary advice, probiotics, and a sham diet. Two studies compared a LFD to non-diet interventions of gut directed hypnotherapy or yoga. The findings clearly support the LFD as an effective treatment in IBS, and although it highlights the role for microbiota and current psychosocial state, it remains challenging to identify what combination of treatments may be best to ensure a personalized approach and overall higher response rates to IBS therapy.

## Introduction

Irritable bowel syndrome (IBS) is a chronic functional gastrointestinal disorder with an estimated worldwide prevalence of 11.2% (1). The condition is characterised by recurrent abdominal pain and altered bowel habits as per the diagnostic Rome IV criteria (1). IBS is associated with decreased quality of life, social productivity, and work performance. Furthermore, IBS not only poses a financial burden to the individual through the cost of seeking medical advice but also impacts the healthcare system by time and resources acquired by patients (2). Nearly 40% of primary care and gastroenterologist visits can be attributed to IBS (3).

Diet, specifically the widely recognized low fermentable, oligosaccharide-, disaccharide-, monosaccharide-, and polyol (FODMAP) diet (LFD), has been a cornerstone therapy for IBS. The LFD involves three phases; a ‘FODMAP restriction phase’ lasting 4–8 weeks, a ‘re-introduction and challenge phase’ lasting 6–10 weeks, and a ‘personalization phase’ where tolerated FODMAPs are returned to the diet (4). Several studies have shown the diet to be efficacious in the management of IBS symptoms (5, 6). However, data still suggest that approximately 30% of individuals do not respond to this management option (7). Furthermore, the safety of the LFD has been questioned in regard to its nutritional adequacy, decreased fiber intake, and potential negative impact on the gut microbiome (8).

The major mechanistic pathways *via* which FODMAPs induce symptoms in IBS are *via* osmotic load and colonic gas production in the setting of visceral hypersensitivity and have been reviewed in depth elsewhere (9). In addition, the gut-brain axis has emerged as an important mechanistic pathway directly modulable through various therapies. This axis is a bidirectional interconnection of the vagal and sacral parasympathetic and sympathetic efferent nerves interacting with the enteric nervous system. The higher brain center can receive signals from the enteric intrinsic, external vagal, and spinal afferents. Dysregulation of this pathway can be attributed to depression, anxiety, and psychological stress (10). Signals relayed between the gut and the brain suggest that IBS is responsive to cognitive and emotive triggers such as stress, anxiety, and depression. Given that psychosocial factors are seen in a high proportion of individuals with IBS, it could explain the refractory response to dietary management of IBS in some patients. Abnormalities in the central sensory processing in IBS patients has become the target for non-dietary related therapies. Psychological therapies (including cognitive therapy and gut-directed hypnotherapy) have shown promise in significantly improving IBS symptoms in adults suffering IBS (11).

Despite several treatment options showing good efficacy for IBS management, particularly in the case of the LFD, there is still much to understand about tailoring the right treatment (whether psychotherapy or diet should be employed as a first line therapy, or a combination of both) to the individual patient. Therefore, the aim of this review was to assess the effectiveness of the LFD compared with non-dietary treatments, in reducing symptoms and improving bowel function, as well as safety considerations such as nutritional adequacy and effects on the colonic microbiota. The findings will provide insight into strengths, limitations, and application of the LFD compared with other dietary and non-dietary interventions, thereby addressing gaps in the literature and future directions for the management of IBS.

## Methods

A literature search was conducted using the Medline, Scopus, Cinahl and Embase databases. Search terms included “irritable bowel syndrome,” “IBS,” “fodmap,” “diet,” “cognitive behavioral therapy,” “complementary and alternative medicine,” “hypnotherapy,” and “herbal medicine.” Intervention studies were included, being either randomized or non-randomized comparative trials that assessed the LFD against another intervention (dietary or otherwise). This inclusion criteria were set so that the LFD could have a clear comparison against another treatment modality. Studies were included if they assessed an adult population, and there was no limitation on year or the therapy the LFD was being compared to. No limitations were placed on IBS subtype. Data reviewed within these studies included diagnostic criteria (Rome III or Rome IV), intervention duration, assessment of symptom measures, changes to gut microbiome, type and overall effectiveness of the intervention implemented (education and resources), gaps in the literature, and future research directions within an IBS population.

## Results

Ten studies were included in this review, which assessed the LFD against other treatments (**Table 1**). Six of these studies compared the LFD against other dietary interventions including traditional dietary advice (12), modified National Institute for Health and Care Excellence (NICE) guidelines (13), high FODMAP diet (14), gluten-free diet and Mediterranean diet (15), generalized dietary advice and (8, 16). Two studies compared the LFD to probiotics (17, 18) and two studies compared a LFD to non-diet interventions—gut directed hypnotherapy (19) or yoga (20). One study (17) used a factorial design with participants allocated to either the shame diet/probiotic, sham diet/placebo, the LFD/probiotics, or the LFD/placebo; however, no interaction effect for symptoms or microbiota changes were noted, so data for the LFD compared to probiotics was not reported. Therefore, only results for the LFD compared to the sham diet have been included in the current analysis.

**Table 1:** Summary of trials reporting on the assessment of the low FODMAP diet compared with other interventions in the management of IBS.

Author, year, country	Study design	Population, diagnostic criteria, and source of recruitment	Intervention and duration	Gastrointestinal symptom and microbial measures	Effect on symptoms	Practice implications
***LFD vs. other dietary interventions***						
Bohn et al. (12) 2015 Sweden	Randomized, multi-center, single blind	n = 67 Adults aged 18–70 years Rome III IBS outpatient clinics	4-week LFD or NICE guidelines (regular meals, reduced fat, fiber, caffeine, and gas reducing foods)	IBS-SSS Bristol stool form scale	Symptoms reduced within both groups (p = <0.00001) but no difference between groups (p =0.2) Mean stool frequency improved significantly within the LFD from baseline to 4 weeks (1.9 ± 0.8) to (1.5 ± 0.7), p = <0.001) as per the Bristol stool form scale. Stool frequency had a non-significant change in the NICE group at baseline) (1.6 ± 0.7) compared to 4 weeks (1.5 ± 0.6), p =0.15). There was a non-significant difference between the groups at 4 weeks (p =0.64)	*Overlap between two diet interventions on reduction in ‘gas-forming foods’ and other components of FODMAPs suggest efficacy favoring LFD^†^* *Potential for ‘sensible’ eating guidelines to have additive effects to LFD*
Eswaran et al. (13) 2016 United States of America	Randomized, single center open label trial	n = 92 Adults aged 18 years and over Rome III (IBS-D subtype) Gastroenterology and primary care clinics	4-week LFD or modified NICE (mNICE) guidelines	11-point likert scale Weekly global symptom assessment Bristol stool form scale	52% LFD vs. 41% mNICE reported adequate relief (p = 0.031) LFD had higher proportion of abdominal pain responders compared with mNICE (51% vs. 23%, p = 0.008) At 4 weeks, stool consistency improved significantly on the LFD compared to the mNICE guidelines (p<0.0001) as per the Bristol stool form scale	*LFD^†^ produced a greater improvement in abdominal pain, bloating, stool consistency, stool frequency and urgency at 1*-week *mNICE guidelines showed no significant improvement in abdominal pain, bloating or stool frequency in any wk* *Compared to baseline, both diets showed improvement for abdominal pain, bloating, stool consistency, stool frequency and urgency at 4-week*
McIntosh et al. (14) 2017 Canada	Prospective, randomized, single blind parallel study	n = 37 Adults aged 18 years and over Rome III Outpatient clinics	3-week LFD or HFD	IBS-SSS 16s RNA profiling	IBS-SSS reduced in LFD but not in HFD (p = <0.001) No differences in α or β diversity between samples from before or after HFD or LFD across IBS subgroups	*LFD^†^ showed greater reduction in abdominal symptoms at 3-week* *HFD led to increased pain at 3 weeks* *Subgroup analysis showed IBS-M and IBS-D participants had higher bacterial richness after the LFD at 3 weeks*
Paduano et al. (15) 2019 Italy	Non-randomized cross over clinical trial	n = 92 Adults aged 18–45 years Rome IV GI outpatient clinics	4-week LFD or gluten-free or Mediterranean diet	IBS-SSS VAS for bloating and abdominal pain Bristol stool form scale	All 3 diets reduced symptom severity (<0.01), bloating (p<0.01) and abdominal pain (p<0.01) The LFD improved stool solidarity from a type 6 to a type 4 (p = 0.03) which was further supported by 79% of LFD participants showing a trend to reach type 4 after 4 weeks on the LFD. No statistically significant differences were observed in stool solidarity for the gluten-free and Mediterranean diets at 4 weeks (data not shown)	*Adequate FODMAP distribution over the day was key to preventing overload of FODMAPs in a single meal and inducing symptoms* *LFD^†^ showed superiority for improving overall & individual GI symptoms, including stool consistency*
Staudacher et al. (8) 2011 United Kingdom	Non-randomized clinical control trial	n = 82 Adults aged 18 years and over NICE criteria Dietetic outpatient clinic follow-ups	36*-*week LFD or standard dietary advice based on NICE guidelines (if a dietitian had already been seen)	16-point VAS scale that included symptoms 7-point Likert scale for symptoms based on IBS global improvement scale	LFD reported greater satisfaction in symptom response (p = 0.38) LFD showed better overall symptom response (p = 0.001), improvement in bloating (p = 0.002), abdominal pain (p = 0.023) and flatulence (p = 0.001)	
Zahedi et al. (16) 2018 Iran	Randomized, controlled single blind trial	n = 110 Adults aged 20–60 years Rome III (IBS-D) Hospital GI clinic	6*-*week LFD or British Dietetic Association guidelines	IBS-SSS Bristol stool form scale	LFD decreased IBS-SSS for abdominal pain intensity (p = 0.001) and frequency (0.017), abdominal distention (p = <0.001), dissatisfaction with intestinal transit (p = 0.001) and interference with daily life (p = 0.005) Mean stool consistency significantly improved in the LFD from baseline to week 6 (5.92 ± 0.45 to 4.3 ± 0.5, p = <0.001) and for the generalized dietary advice group from baseline (5.67 ± 0.61) to week 6 (4.61 ± 0.69, p = <0.001) Mean stool frequency significantly improved in the LFD from baseline to week 6 (3.29 ± 0.87 to 1.91 ± 0.56, p = <0.001) and for the generalized dietary advice group from baseline (3.3 ± 0.77) to week 6 (2.6 ± 0.96, p = <0.001)	*Both diets reduced symptom severity* *LFD compared to generalized dietary advice decreased symptoms for each subset of IBS-SSS and produced relief of symptoms at each timepoint (baseline, 3 weeks, and 6 weeks)* *Both diets improved stool frequency and consistency at 6 weeks*
Staudacher et al. (17) 2017 United Kingdom	Randomized, Double-blind 2x2 factorial design	n = 104 Adults aged 18–65 years Rome III Tertiary hospitals	4*-*week LFD or sham diet and placebo or multi-strain probiotic formulation	Gastrointestinal symptom rating system (GSRS) IBS-SSS Bristol stool form scale qPCR and 16sRNA sequencing	A higher proportion of patients on LFD had adequate symptom relief than sham diet (p = 0.042) LFD showed lower IBS-SSS score than sham diet (p = 0.01 but not different between probiotic and placebo (p = 0.721) LFD showed a higher proportion of participants achieved clinically meaningful reduction of >50-point reduction in total IBS-SSS compared to sham diet (73% vs. 42%) There was a significant difference in mean stool consistency at 4 weeks between the sham diet (4.3 ± 1.1) compared to the LFD (3.9 ± 1.0), p = 0.008 as per the Bristol stool form scale. The was no significant difference for the placebo and probiotic group for stool consistency (4.2 ± 1.0 vs. 4.0 ± 1.1), p = 0.544, respectively At 4 weeks here was lower absolute *Bifidobacterium* species abundance in LFD compared to sham diet (8.8 16s rRNA genes/g (SD 0.6) vs. 9.2rRNA genes/g (SD 1.0) mean difference -0.39 rRNA genes/g (95% CI, -0.64 to -0.13, p = 0.008) and greater abundance of *Bifidobacterium* species for probiotic compared to placebo [9.1 rRNA genes/g (SD 0/6) vs. 8.8 rRNA genes/g (SD 1.0) mean difference +0.34 rRNA genes/g (95% CI, 0.05 to 0.61, p = 0.019]	*LFD^†^ showed greater efficacy in improving GI specific and overall symptoms compared to sham dietary advice at 4 week* *LFD-induced effects on microbiota can be modified with adjunct probiotic therapy*
***LFD vs. probiotics***						
Pederson et al. (18) 2014 Denmark	Randomized, open label control trial	n = 123 Adults aged 18–74 years Rome III Tertiary hospital	6*-*week LFD or normal diet (ND) or lactobacillus rhamnoses GG probiotic (LGG)	IBS-SSS	LFD reduced IBS-SSS from baseline to 6 weeks compared to LGG vs. ND (p = <0.01) IBS-SSS scores reduced in LFD and LGG group compared to the normal diet (133 ± 122 vs. 68 ± 107, 133 ± 122 vs. 34 ± 95, p = <0.01) at 6 weeks	*LFD superior over probiotic alone across all IBS subtypes except IBS-C*
***LFD vs. non-dietary interventions***						
Peters et al. (19) 2016 Australia	Randomized open-label, parallel study	n = 74 Adult aged 18 years and over Rome III General IBS population	6*-*week LFD or gut-directed hypnotherapy or a combination of both	100 mm VAS for symptoms (abdominal bloating, wind, abdominal pain, nausea, and satisfaction with stools)	Improvements in all symptoms were observed from baseline to 6 weeks for hypnotherapy, LFD and combination treatment with no difference across groups (p = 0.67)	*While both gut-directed hypnotherapy and LFD were equally efficacious in the short (6 weeks) and longer term (6 months), gut-directed hypnotherapy showed a greater benefit on psychological indices compared to LFD* *Combining two equally efficacious therapies did not necessarily confer added benefits for IBS patients*
Schumann et al. (20) 2018 Germany	Randomized, single blind study	n = 59 Adults aged 18–75 years Rome III Online and local press, department of internal and integrative medicine	12*-*week LFD or yoga	IBS-SSS	No significant differences between groups regarding IBS-SSS, except for abdominal distention subscale at 12 weeks (p = 0.040) in favor of LFD IBS subtype analysis showed no significant differences between interventions for effectiveness (data not shown)	*LFD^†^ showed higher proportion of participants who achieved clinically meaningful reduction in IBS-SSS at 12 weeks* *Clinical remission was sustained in equal number of patients between both groups at 6-month follow-up*

GI, gastrointestinal; GSRS, gastrointestinal symptom rating scale; HFD, high FODMAP diet; IBS-C, irritable bowel syndrome-constipation, IBS-D, irritable bowel syndrome-diarrhea; IBS-M, irritable bowel syndrome-mixed; IBS-SSS, irritable bowel syndrome symptom severity score; NICE, National Institute for Health and Care Excellence; VAS, visual analogue scale; ^†^indicates the LFD was superior for treatment response.

This review included studies with a range of comparative study methodologies. All except two studies were randomized controlled trials (RCTs) (8, 15). Of these studies, four were single-blind RCTs (12, 14, 16, 20), one was double-blind (17), with the remainder being open-label (13, 18, 19). Four studies (8, 13, 18, 19) were adequately powered (12, 14–18, 20). Data for IBS subtypes were not available for all studies, but where available (8, 12, 14, 17–19), the evidence has been discussed in relation to its applicability to the specific subtype.

### LFD vs. Other Dietary Treatments

#### Symptom Severity

Five studies (12, 14–17) used the IBS-SSS to compare symptom severity in a LFD compared to other dietary treatment. The IBS-SSS is a five-item questionnaire scored using a VAS. One study (12) found IBS-SSS improved symptoms in each group with no significant difference between groups (p = 0.20). In the remaining studies, the LFD demonstrated superior efficacy in reducing IBS-SSS scores in comparison to traditional dietary advice, a high FODMAP diet (HFD), a gluten-free diet, generalized healthy eating, and a sham diet.

Two studies (8, 13) used scoring systems other than the IBS-SSS to assess changes in symptoms. For both studies, the LFD had a greater reduction in symptoms overall at the end of intervention compared to baseline after a minimum of 4 weeks. One study (8) showed a greater reduction for each individual question and globally with a composite score of the questions on the LFD.

#### Bristol Stool Form Scale

Five studies assessed bowel habits using the Bristol stool form scale as a measure of stool consistency and frequency (12, 13, 15–17). Overall, there was a trend toward the LFD improving stool consistency (13, 17) and frequency (16). One study reported the LFD having the greatest improvement in IBS-D subtype (15).

None of the remaining interventions produced an effect on stool form or number of bowel motions (12, 15).

#### IBS Subtype Response to Treatment

The IBS-D subtype showed a positive response to the LFD at 1 week (13) and 6 weeks (16). One study (14) showed that at 4 weeks, the LFD had the following changes; 14 of 16 IBS-D showed an improvement in bowels, 7 of 10 with IBS-C showed bowel improvement, and of eight with IBS-M, two participants showed improvement, one worsened, and two had no changes. The remaining participants were all IBS-U, undefined at baseline (15). These findings suggest that the LFD benefits each IBS subtype, most consistently for IBS-D.

#### Delivery of Dietary Intervention

Given the LFD approach is comprehensive due to the elimination, reintroduction, and personalization of the diet, there are potential risks if the diet is not implemented safely. Alterations to gastrointestinal microbiota and nutritional adequacy have been noted after just 4 weeks of a LFD, which is concerning given that the initial restriction phase is usually 6 weeks (4). Personalized dietary advice from a dietitian has been positively associated with compliance and success (21). In all studies evaluating the LFD compared to other dietary intervention, it was promising to see all involved the expertise of a dietitian in delivering the LFD diet (8, 12–16). Additionally, a major factor in determining the success of the LFD was the provision of written resources to facilitate implementing the diet (22). There were varying degrees of contact with the dietitian where some participants received 45 to 90 min on a single occasion or up to four sessions in either an individual or group setting. In some studies, there was limited contact with the dietitian to replicate clinical practice. Commonly noted feedback to study personnel were that participants found the diet relatively easy to follow, but the translation of low FODMAP foods into recipes was difficult. One study, which had the low FODMAP food resource prepared in accordance with Iranian culture, found that adherence was considered difficult; however, it was not reported as a problem in the trial (16). Adherence with the LFD was associated with achieving a clinically important value of a reduction in IBS-SSS ≥50 (12).

For the other dietary interventions, there was insufficient detail provided to ascertain whether participants received the same level of care as those receiving a LFD. Therefore, the quality of dietetic care is less comparable to those who received LFD intervention, and there is insufficient insight what participants were specially instructed to do to elicit symptomatic relief.

#### Effects on Microbiome

##### Overall Analysis

Changes in the dietary content of fermentable carbohydrates have previously been shown to have a major influence on the gut microbiota composition. Alpha and beta diversity were not different after the implementation of a HFD or LFD from baseline to end of intervention and the result was consistent across IBS subtypes (14). There were no significant differences in the alpha diversity for the LFD compared to the sham diet (p = 0.401). The LFD compared to the sham diet did not produce a difference in beta diversity either (p = 0.575) (17). At a taxonomic level, the genus *Aldercreutzia*, *Dorea*, and the family Actinomycetaceae were lower after following a HFD (p = 0.02, p = 0.05, and p = 0.04, respectively). However, after just 3 weeks, the LFD produced fecal samples with higher Actinobacteria richness and diversity compared with the HFD group (p = 0.046 and p = 0.02, respectively) (14). Several bacterial groups decreased after following the HFD, with the exception of the Bifidobacteriaceae family and unclassed family within the Lachnospiraceae family, which increased (14). On a species level, the LFD compared with the sham diet produced lower absolute abundance of *Bifidobacterium* (p = 0.08). The LFD did not produce a difference in relative abundance of the *Streptococcus* species or the *Lactobacillus* species compared to the sham diet between baseline and follow-up (17). These findings suggest that the alpha and beta diversity may not be impacted by the implementation of a LFD; however, at a species level, the results are inconsistent.

##### Subgroup Analysis

When the IBS subtypes IBS-M and IBD-D (both groups having some diarrhoea) were analyzed, there was a greater bacterial richness in those following the LFD compared to the HFD (p = 0.047). Actinobacteria diversity was increased (p = .013), and Firmicutes, Clostridiales, and Actinobacteria richness was greater (p = 0.029, p = 0.023, and p = 0.029, respectively) (14).

### LFD vs. Probiotics

#### Symptom Severity

Symptom severity was measured using the GSRS (17). The LFD produced a 117-point decrease on the IBS-SSS compared to the probiotic with an 82-point decrease. Probiotics did not produce a statistically significant overall symptom improvement using the GSRS (p = 0.66), but the LFD was significant (p = 0.020) (17).

#### Bristol Stool Form Scale

Pedersen et al. did not use the Bristol stool form scale.

#### Delivery of Dietary Intervention

One study compared a LFD to probiotic use whereby participants were seen by either a dietitian or nutritionist (18). Dietary counselling was provided for up to one hour (18) with a complex list of appropriate foods provided by a dietitian (18). Dietary compliance was regularly checked and contact with the dietitian was encouraged (18). Probiotics were administered in capsules (18) whereby participants consumed two capsules each day.

#### Effects on Microbiome

Data on the LFD and probiotic on microbiome was not reported (18).

#### IBS Subtype Response to Treatment

The IBS-D subtype showed a positive response to the LFD at 6 weeks (18). In addition, IBS-M subtype showed a positive response to the LFD and LGG probiotic at 6 weeks. The IBS-C subtype did not have a positive response to the LFD, probiotic intervention, or a normal diet (18).

### LFD vs. Non-Dietary Treatments

#### Symptom Severity

For two studies comparing a LFD to non-dietary treatments, there was a significant decrease in symptoms for the LFD from baseline to end of intervention (p < 0.001) (19) and (p < 0.001 and p < 0.001 for yoga and a LFD, respectively) for IBS-SSS scoring (20). For both studies, there were no significant differences between the groups at baseline compared to end of intervention.

#### Bristol Stool Form Scale

Neither of the studies that assessed non-dietary interventions used the Bristol stool form scale as an outcome.

#### Delivery of Intervention

The participants receiving gut-directed hypnotherapy were allocated 1 h weekly sessions throughout the 6-week study duration. Each participant received the same script that was also recorded and given to the participants to listen to daily for the duration of the study. The intervention was provided by an experienced clinical hypnotherapist (19).

Participants who received the yoga intervention had twice weekly group sessions, which were 75 min in duration for a 12-week period. The classes were guided by the same certified hatha yoga instructor. Specifically, participants were instructed on customised postures and breathing techniques to improve symptom control (20).

#### IBS Subtype Response to Treatment

There were no differences in treatment effectiveness between IBS subtypes (19, 20).

## Discussion

This review highlights that the LFD is efficacious in the management of IBS. Despite its success, several considerations need to be addressed regarding its use. While there has been greater understanding of the LFD and its mechanism in practice with recent research, there is still a consensus that further understanding of the diet’s implications are needed.

Gaps of interest include a deeper understanding on the long-term effects of the LFD on gut microbiota diversity. It should be established whether a change in the microbiota profile can be attributed to a mediated symptom response (8, 14). Although recent reviews indicate that baseline microbiota may not be an accurate predictor of symptom improvement in IBS (23), the volatile organic compound profile may very accurately select responders, suggesting that understanding metabolic function of bacteria is more important for determining response to dietary interventions (24). Modulation of gut microbiota with the use of pre- and probiotics while implementing the LFD should be considered. While prebiotics can infer a symptomatic response in some individuals, it should be ascertained whether a less restrictive LFD mitigates the negative impact on the microbiota.

From a safety perspective, calorie and nutrient inadequacies have been acknowledged when following the LFD. Therefore, excessive restrictions such as the avoidance of complete food groups should be averted (25). The implementation of the LFD is extensive and requires education from a qualified nutrition professional (4). Where the LFD may not be appropriate or possible, other dietary strategies can be considered. Evidence suggests that simple strategies such as a reduction in gut stimulants (caffeine, alcohol, and spicy food) and modulation of meal size and frequency may also be effective. However, it should be noted that the NICE guidelines include recommendations such as reducing polyols, onions, cabbage, and beans and limiting fruit to three portions per day. Despite being considered as generalized dietary advice, these foods contain FODMAPs, which is why a reduction in symptoms may be concurrent if following this advice. Furthermore, dietitians instructed participants in one study who were not randomized to the LFD to limit consumption of foods that contribute to perceived detrimental symptoms (16). While the dietitians did not advise these participants to restrict FODMAPs specifically, it is unknown if any foods restricted did contain FODMAPs, which could have contributed to symptom improvement. From a practical perspective, it appears there is still a need for data on the LFD when followed for a long duration.

Cognitive considerations in IBS also warrant further investigation. The mechanisms in which gut directed hypnotherapy exert an effect on the gut are not fully understood but suggest the control and normalisation of gastrointestinal function can be made to the subconscious mind. Peters et al. found that improved psychological indices were not correlated with symptomatic benefit, although the study was not designed to evaluate mechanisms for efficacy; therefore, further understanding the mechanisms of gut hypnotherapy on symptom improvement is needed (19).

Despite results from Peters et al. that a combination of dietary and psychological interventions showed no additive benefits, areas for further research would be further exploring combining dietary and other non-dietary interventions. Tailored advice based on an individual’s current dietary intake and other psychosocial factors should help to inform a management plan. Given that the gut microbiota have an established role in IBS and the gut-brain axis (26), combined dietary and cognitive treatments should be examined to determine the relationship between concurrent changes to the gut microbiota and symptom resolution.

This review highlighted a heterogeneity in LFD study designs and in IBS patient selection, including a lack of inclusion or reporting of specific IBS-subtypes. Regardless, the findings demonstrate consistencies in the evidence that the LFD is efficacious in overall symptom and bowel function improvement for each IBS subtype, allowing practical application across the distribution of IBS patterns.

In conclusion, the LFD is efficacious in reducing symptoms when compared to other dietary and non-dietary treatments, however it remains difficult to understand why some individuals respond to certain treatments while others do not. Future research should focus on identifying which treatment modality specifically or in which combination *via* a multimodal approach is best suited to an individual with IBS, including short- and long-term effects. Current dietary intake and symptom pattern of an individual in conjunction with current psychosocial state regarding depression, anxiety, and stress should be measured to best inform whether dietary or cognitive therapies are likely to be more effective in the management of IBS.

## Author Contributions

Conceptualization: LM and JB. Writing and draft preparation: LM. Review and editing: CY and JB.

## Conflict of Interest

CY has received research support from Yakult Australia and Danone Australia for investigator-initiated studies.

The remaining authors declare that the research was conducted in the absence of any commercial or financial relationships that could be construed as a potential conflict of interest.
